# Exploring the Psychological Experiences of Patients With Melanoma: A Narrative Review

**DOI:** 10.7759/cureus.79637

**Published:** 2025-02-25

**Authors:** Saajan Bhakta, Lisa Brambert, Grace Bartlett, Sainamitha R Palnati, Ritwick S Mynam

**Affiliations:** 1 Department of Research, Kansas College of Osteopathic Medicine, Wichita, USA; 2 Department of Medicine, University of Wisconsin School of Medicine and Public Health, Madison, USA

**Keywords:** advanced melanoma, mental health services, psychological stressors, psycho-oncology, unresectable melanoma

## Abstract

The increased prevalence of psychological stressors in individuals diagnosed with melanoma, compared to the general population, is well-documented. Understanding the psychological experiences of patients with advanced unresectable stage 3 or 4 melanoma is essential for providing holistic treatment and supporting the unique physical, emotional, and mental health needs of this population. Literature searches were conducted in MEDLINE, PubMed, and Google Scholar to identify studies that focused on the psychological experiences of patients with unresectable stage 3 or 4 melanoma, resulting in a narrative review. Records (n=482) were screened to include peer-reviewed studies published in the last 25 years, primary research involving melanoma patients, or systematic reviews and meta-analyses involving melanoma and mental health. The studies reviewed (n=13) consistently observed psychological changes in melanoma patients, with many reporting higher rates of anxiety and depression compared to the general population. Despite these findings, research on advanced melanoma and its psychological impact remains diverse but lacks specificity regarding the experiences of patients with unresectable stage 3 or 4 melanoma. While valuable insights have been gained into the assessment tools, prevalence, and potential treatments for psychological stressors, no study has explored these psychological experiences in-depth from the patient's perspective. Further investigation into the psychological experiences of this patient group is critical to improving psychological support and fostering more comprehensive care for oncology patients.

## Introduction and background

Melanoma is a cancer originating in the skin's melanocytes, which has the ability to metastasize to other areas of the body [1]. Psychological evaluation is an essential component of comprehensive care for all patients, particularly those facing chronic, life-altering, and potentially terminal diagnoses such as metastatic melanoma. Current systematic reviews have shown a higher prevalence of anxiety and depression in individuals with melanoma compared to the general population [2,3]. The National Institute of Mental Health estimates that among US adults, 19.1% have anxiety and 8.3% have depression [4,5]. Kasparian et al. reported that 30% of patients with a melanoma diagnosis experienced clinical psychological distress [2]. This finding is supported by a systematic review and meta-analysis by Danielsen et al., which cited that 6%-16% of this population experienced depression, and 7%-30% experienced anxiety across all stages of treatment [3]. This discrepancy was evident in patients' scores on validated instruments such as the Hospital Anxiety and Depression Scale (HADS) and the European Quality of Life-5 assessment. Additionally, interferon immunotherapy for melanoma has known depressogenic effects [6], and biological markers, such as cytokine elevations and gene mutations, have been associated with increased psychological distress in these patients [7,8]. While Kasparian et al. recommended developing a risk factor screening tool to identify and support melanoma patients most susceptible to psychological distress, no such tool has been developed as of this review [2]. Although studies have shown promising outcomes with cognitive behavioral therapy (CBT) [9] and psychosocial support groups [10] for this population, there is limited focus on late-stage melanoma patients, particularly those with stage 3 or 4 unresectable (irremovable by surgery) melanoma. These patients are not candidates for surgical cancer removal due to tumor thickness and metastasis [1]. This review examines the current literature on psychological considerations for melanoma patients, highlighting the insufficient attention given to the late-stage population and the lack of an appropriate assessment tool for addressing their psychiatric needs.

## Review

Methods

**Figure 1:**
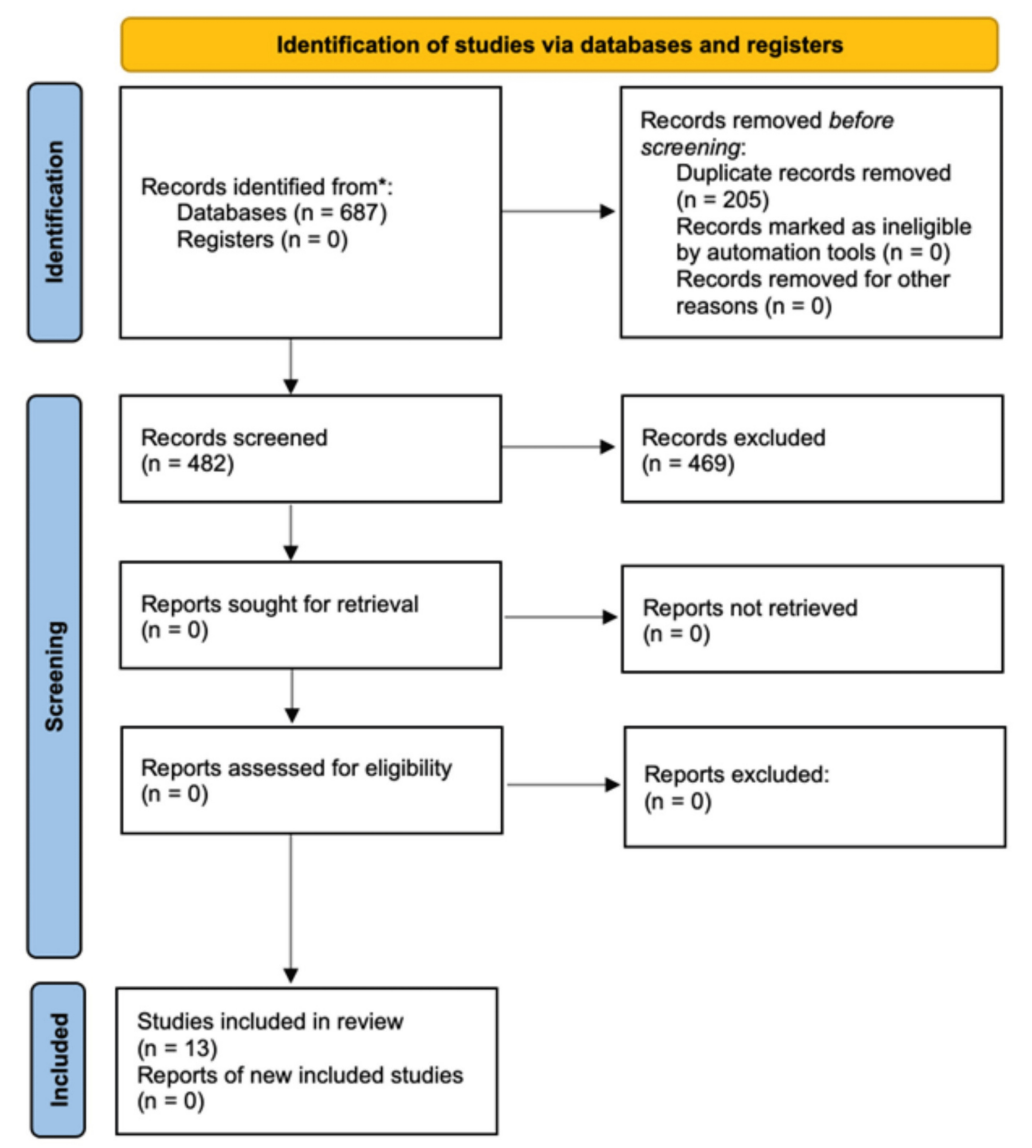
PRISMA flow diagram of the summarized search strategy n: number of studies, PRISMA: Preferred Reporting Items for Systematic Reviews and Meta-Analyses

To develop this narrative review, electronic databases were searched from April 18, 2024, to May 2, 2024, including Google Scholar, PubMed, and MEDLINE. The search utilized keywords and phrases such as melanoma, unresectable melanoma, stage 3 melanoma, stage 4 melanoma, metastatic melanoma, mental health, anxiety, depression, psychology, psychiatry, stressors, experiences, quality of life, and well-being. Boolean operators were employed between keywords to ensure a comprehensive search. This includes but is not limited to the following: melanoma and anxiety or depression, melanoma and depression, melanoma and anxiety, unresectable melanoma and mental health, and so on. The inclusion criteria for this review were peer-reviewed studies published in the last 25 years, primary research involving melanoma patients, or systematic reviews and meta-analyses involving melanoma and mental health. The exclusion criteria included studies unrelated to melanoma and mental health, case reports, non-peer-reviewed studies, and non-English publications. All records were independently screened for inclusion by five members of the research team, which yielded a comparative summary of the research studies included in this review (Table 1). Thirteen relevant publications from the 2003-2023 timeframe were included and synthesized into the following sections: (i) psychological experiences, (ii) coping strategies, and (iii) interventions. Figure 1 depicts the summarized search strategy employed for this narrative review.

**Table 1:** Comparative table of the included research studies N/A: not available, HADS: Hospital Anxiety and Depression Scale, COVID-19: coronavirus disease 2019

Study	Study design	Population considered	Number of patients/participants	Time frame	Main findings and outcomes	Limitations
Kasparian et al. [2]	Systemic review	Malignant melanoma patients	N/A	1998-March 2008	The diagnosis of melanoma triggered responses of anxiety, distress, fear of cancer recurrence, and body image concerns among patients. The most effective coping strategies were seeking social support and problem-solving through tailored interventions.	Various studies included in this review presented inconsistent findings, low-quality evidence, and weak recommendations.
Danielsen et al. [3]	Meta-analysis	Melanoma patients	12,400	1992-2022	Anxiety was most evidenced before and after treatment for patients. Depression was most prevalent during treatment for patients.	The studies included in the meta-analysis only focused on depression and anxiety, with a lack of reported data on other components of patients' psychological experiences. Specific subtypes of anxiety and depression were not reviewed.
Kovàcs et al. [6]	Prospective cohort study	Melanoma patients	127	N/A	Low-dose interferon-alpha treatment was shown to induce depression in patients. However, strong social support served a protective role against these depressive symptoms during interferon-alpha treatment.	The open-label design of the study lacked an untreated control group. The study's 12-month follow-up period limited the assessment of long-term psychological and therapeutic effects.
Kim et al. [7]	Cross-sectional study	Acral and non-acral melanoma patients	151	September 2020-March 2021	Pro-inflammatory cytokines showed some correlation with higher psychological distress, although not statistically significant.	The prevalence of melanoma served as a limitation, resulting in sample size constraints. There is a need for further research on late-stage metastatic melanoma.
Rogiers et al. [8]	Cross-sectional study	Metastatic melanoma survivors	104	2016	Survivors of melanoma reported low anxiety and depression. However, emotional distress was evidenced among most survivors.	The study design allowed for analysis of only one point in time. The small sample size lacked the opportunity for generalizability.
Lynch et al. [9]	Prospective cohort study	Metastatic melanoma patients	61	February 2019-June 2019	In-person therapy yielded a reduction in fear of cancer recurrence and fear of progression among patients. Patients who engaged in self-management techniques had expressed a decrease in fear of cancer recurrence.	There was a small sample size, no control group present, and a lack of specificity in screening measures and cut-off scores.
Fawzy et al. [10]	Prospective cohort study	Stage 1 malignant melanoma patients	68	N/A	There was no statistically significant difference between the control and interventional groups at the 10-year follow-up. Factors such as male gender and greater Breslow depth were significant for recurrence and survival, predictive of poorer outcomes.	The study was conducted with a small sample size.
Beesley et al. [11]	Prospective cohort study	High-risk primary cutaneous melanoma patients	675	2010-2014	Anxiety and depression-related symptoms decreased over time since the time of diagnosis for most patients. Factors such as female gender, age, and baseline depression and anxiety levels were found to be associated with persistent distress.	The investigators shared that they may have identified some false-positive and false-negative cases with the use of HADS. The self-reporting nature of the study may have led to underestimation of anxiolytic or antidepressant medication use among patients.
Wang et al. [12]	Cross-sectional study	Unresectable stage 3 and stage 4 melanoma patients	273	February 2020-November 2021	Many patients, who expressed an increased fear of progression, had elevated anxiety and depression. Anxiety and depression levels did not regress over time after diagnosis.	This study was conducted during the COVID-19 pandemic, which may have influenced the psychological experiences of patients.
Miniati et al. [13]	Narrative review	Uveal melanoma patients	2,587	1993-2018	Anxiety levels peaked during diagnosis, treatment, and follow-up visits. Depression was most evident post-treatment in association with long-term effects. Patients with greater social support reported better psychological outcomes.	Studies incorporated in this narrative review had evidence of generalization of study results due to the wide range of instruments used to assess patients' quality of life.
Dunn et al. [14]	Systemic review	Metastatic melanoma survivors	N/A	1992-2015	Higher levels of distress, anxiety, and depression were reported by patients with advanced melanoma (stage 3 and stage 4 melanoma patients). The fear of disease progression was often expressed by patients. Effective psychological support benefited patients.	The studies included in this review had small sample sizes, non-representative sampling, inconsistent measurement approaches, and overall, a lack of theoretically sustainable study designs.
Karamanis et al. [15]	Retrospective cohort study	Women diagnosed with anorexia nervosa and cancer	6,009	1973-2003	There was no statistically significant difference in cancer incidence among women in the general population and women with anorexia nervosa. There was an increased mortality from melanoma in patients with anorexia nervosa, which led to a worse prognosis than compared to the control group.	The study design did not offer adequate statistical power. Data on external factors such as smoking, alcohol consumption, and environmental exposure were not accounted for in this study.
Kan et al. [16]	Randomized controlled trial	Stage 0-2 melanoma patients at high risk of developing new primary disease	183	N/A	A majority of patients were highly satisfied with both psycho-educational resources and intended psychotherapy sessions, as evidenced by the decrease in fear of cancer recurrence in both interventions. Patients benefited from enhanced patient-physician communication, open conversations with family members about melanoma, and improved coping strategies.	Possible recall bias may have affected the accuracy of survey responses since patients completed the survey six months after receiving psycho-educational resources. Information on potential patterns in psychotherapy sessions' content and their effect on intervention outcomes was not assessed.

Literature review

It is critical to thoroughly evaluate the psychological experiences of advanced-stage melanoma patients. This review focuses on three key areas: stressors, coping skills, and interventions. First, the psychological stressors unique to patients with advanced melanoma will be examined, including the emotional and mental challenges they face throughout their diagnosis and treatment. Next, the coping strategies employed by these patients to manage their condition will be explored, highlighting both adaptive and maladaptive responses. Finally, the review will address the various interventions available to support patients' psychological well-being, discussing the effectiveness of current treatments and identifying potential areas for improvement. This structure aims to provide a comprehensive understanding of the psychological experiences of this patient population.

Psychological Experiences

As part of the Primary Melanoma Project in Queensland, Australia, Beesley et al. studied anxiety and depression in individuals diagnosed with high-risk primary cutaneous melanoma between 2010 and 2014 [11]. With a majority of male patients (n=392, 58%), this longitudinal study of 675 patients (mean=62 years old, standard deviation (SD)=14) used the standardized Hospital Anxiety and Depression Scale (HADS) for assessing anxiety and depression over a four-year time period. Patients who demonstrated significant anxiety and depression at the time of diagnosis generally decreased over time [11]. At the time of diagnosis, 93 (14%) patients displayed melanoma-related anxiety or depression. After about six months, this decreased to about 13-34 patients (2%-5%) who remained recurrence-free. While this suggested that the fear of recurrence is short-term, persistent new-onset anxiety or depression was evidenced among 39 (7%) younger patients who were aged <70 years old. By contrast, those with anxiety and depression related to other factors stayed consistent throughout this time period, at about 108-142 (16%-21%) patients. It was found that female gender, young age, and higher levels of depression and anxiety at baseline were associated with persistent distress. The authors noted that social support and the development of effective coping mechanisms alleviated some psychological distress [11].

The COVID-19 pandemic was an unprecedented time for both medical professionals and the general public. Wang et al. utilized a cross-sectional survey study at Xijing Hospital of the Fourth Military Medical University in China to investigate fear of progression, depression, and anxiety in 273 stage 3 or unresectable melanoma patients recruited between February 2020 and November 2021 [12]. Standardized surveys were utilized to assess these factors such as the Fear of Progression Questionnaire-Short Form (FoP-Q-SF) (Cronbach α=0.883), State-Trait Anxiety Inventory (STAI-6) (Cronbach α=0.77-0.83), and Patient Health Questionnaire (PHQ-9) (Cronbach α=0.89-0.92). The authors found that 174 (64.7%) of those surveyed reported increased fear of progression (mean=39.9, SD=11.0), 198 (72.5%) patients experienced anxious symptoms (mean=13.1, SD=3.0), and 62 (22.7%) patients had elevated depression (mean=6.4, SD=6.1) [9]. Anxiety and depression were significantly positively correlated with each other (r=0.309, p<0.001). Finally, clinical anxiety was associated with cancer stage (p=0.001), and cancer recurrence or progression (odds ratio (OR)=14.394, p<0.001) were more likely to report higher anxiety levels [12]. A vital finding unique to this study compared to other similar ones was the lack of anxiety and depression regression over time post-diagnosis. The authors speculated that the complications set forth by the COVID-19 pandemic such as fear of infection with SARS-CoV-2, delays in treatment, and uncertainty of the future contributed to these unique psychological experiences [12]. This finding emphasized the importance of continuous psychological patient support. Limitations presented by this study design include its sample size, homogeneous population, and cross-sectional methodology. There is also increased potential for confounding bias, as this study occurred during the pandemic when anxiety and depression were elevated in the general population.

Danielsen et al. performed a systematic review and meta-analysis of the literature regarding the impact of psychological and behavioral symptoms in melanoma patients [3]. This included 66 studies published between 1992 and 2022 and a total of 12,400 melanoma patients. Studies were included if they reported quantitative data on symptoms such as anxiety, depression, distress, fear of progression, and coping strategies. Statistical analysis was used to quantify the prevalence rates of various psychological and behavioral symptoms across included studies. Quantified by HADS, the prevalence of anxiety was observed in 868-3,720 melanoma patients (7%-30%) and depression in 744-1,984 melanoma patients (6%-16%). Studies showed that anxiety was most prevalent before treatment (n=1,308, K=9; 95% confidence interval (CI)=0.11-0.30) and after treatment (n=6,830, K=26; 95% CI=0.13-0.19). The highest rate of depression was evidenced during treatment (n=71, K=10; 95% CI=0.06-0.22). The authors noted that studies investigating anxiety and depression in melanoma populations had the most robust data for close examination [3]. None of the studies regarding cognition fulfilled the inclusion criteria to be included in the meta-analysis, displaying the need for additional research on broader psychological topics such as fear of progression, sleep, cognition, and fatigue.

The review article by Miniati et al. focused on the quality of life, depression, and anxiety among patients with uveal melanoma (UM) from 18 publications over the last 25 years [13]. Patients with UM tended to experience diminished quality of life related to vision impairment, fear of disease recurrence or progression, and the burden of treatment. High levels of depression and anxiety were prevalent among patients with UM, largely stemming from concerns about the prognosis, potential vision loss, and the risk of metastasis. All uveal melanoma patients assessed by Medical Outcomes Study 36-Item Short-Form Health Survey (MOS-SF-36) and Beck Depression Inventory instruments reported some level of depression one year post-surgery, with 14 of 16 (87.5%) patients falling in the "minimum" range and two of 16 (12.5%) patients scoring "mild." In the cutaneous melanoma population, HADS and STAI-B were employed to demonstrate that 56% of the sample experienced moderate anxiety [13]. Anxiety often spiked during diagnosis, treatment, and follow-up phases, while depression tended to be more associated with long-term effects such as vision loss or metastatic disease. Psychological well-being was influenced by factors such as age, gender, disease stage, and social support. Patients with greater social support, fewer symptoms of vision impairment (p<0.001), and a better understanding of their disease tended to report better psychological outcomes. Miniati et al. noted a statistically significant decrease in anxiety and depression one month after treatment (before treatment: 8.45±4.23, after treatment: 7.33±4.43; p<0.009) [13]. The review emphasized the importance of psychosocial interventions for UM patients, particularly interventions aimed at improving coping skills, managing anxiety and depression, and enhancing quality of life. Counseling, psycho-education, and support groups were noted as effective ways to help patients deal with the psychological and emotional challenges associated with their disease. Psychological support and tailored interventions are critical to help these patients maintain their mental health and improve their overall quality of life [13]. This article highlighted the need for comprehensive care that includes addressing the mental health and quality of life of uveal melanoma patients, in addition to managing the physical aspects of the disease.

Dunn et al. focused on the psychosocial outcomes for patients with advanced melanoma. Advanced melanoma, characterized by metastasis beyond the skin, presented significant challenges not only medically but also psychologically. Dunn et al. aimed to consolidate findings on the psychosocial impact of advanced melanoma, including mental health outcomes and quality of life. Patients with advanced melanoma often experienced high levels of psychological distress, including anxiety (28% of stage 3 melanoma patients and 20% of stage 4 melanoma patients) and depression (19% of stage 3 melanoma patients and 16% of stage 4 melanoma patients according to HADS). This distress is exacerbated by the uncertainty of prognosis and the severity of the disease. The quality of life in these patients is generally compromised due to the physical symptoms of the disease, side effects of treatment, and emotional strain. Factors such as pain, fatigue, and reduced functional status contributed to lower overall well-being. The prevalence of depression and anxiety was notably high among patients with advanced melanoma. Depression can be particularly severe, impacting both emotional and physical health. Patients frequently experience a fear of disease progression and concerns about the future, which contribute significantly to their psychological distress [14].

Kim et. al conducted a cross-sectional study focused on acral and non-acral melanoma and proposed potential pathophysiology for increased psychological distress among this population. They examined plasma cytokine levels and gene mutations and drew correlations between these markers and psychological dysregulation [7]. The psychological symptoms were evaluated via HADS with the anxiety and depression subscales. This study included 151 melanoma patients in total, of which 22 (14.6%) patients had anxiety (mean=4.3, SD=2.7) and 45 (29.8%) patients had depression (mean=5.2, SD=3.8). Pro-inflammatory plasma cytokines, which showed a correlation with higher psychological distress, included interleukin (IL)-2, IL-12, tumor necrosis factor-α (TNF-α), and interferon-gamma (IFN-γ). Anti-inflammatory markers of correlation included IL-4, IL-5, and IL-10. However, Kim et al. noted that there was no statistically significant correlation between cytokine levels and psychological distress (anxiety, depression, and quality of life). They examined the catechol-o-methyl transferase (*COMT*) gene mutation in this population due to its previously shown correlations with anxiety and depression. They found that *COMT* gene mutation exhibited a significant correlation with a diagnosis of acral melanoma. Kim et. al cited the rarity of melanoma as a barrier to stratifying a sample that exhibits consistent type, treatment, and stage of treatment [7]. Although it will result in sample size constraints, this reiterates a need for further investigation of more specific groups such as those with late-stage metastatic melanoma.

Coping Strategies

Dunn et al. underscored the importance of integrating psychosocial care into the overall treatment plan for patients with advanced melanoma [14]. This included regular screening for mental health issues and providing appropriate psychological support. Personalized care plans that address individual psychological needs and preferences are recommended to enhance patient outcomes. Dunn et al. concluded that patients with advanced melanoma experience significant psychosocial challenges, including high levels of distress, depression, and anxiety, which negatively affect their quality of life. Effective psychosocial support and tailored interventions are crucial for improving mental health outcomes and overall well-being in this patient population. This review highlighted the need for comprehensive care that not only addresses the physical aspects of advanced melanoma but also provides robust psychological and emotional support [14].

Rogiers et al. conducted a cross-sectional study to evaluate neurocognitive function, psychosocial outcomes, and quality of life in first-generation metastatic melanoma survivors treated with ipilimumab [8]. The methodology consisted of neurocognitive testing, psychosocial evaluations, and health-related quality of life (HRQoL) assessments. Survivors demonstrated no neurocognitive impairment; ipilimumab treatment did not adversely affect cognition. Survivors had low anxiety and depression. HRQoL was generally high among survivors. However, semi-structured clinical psychiatric interviews indicated persistent disease-related emotional distress among 53% of survivors, which suggested the need for emotional coping strategies [8]. Notable limitations of this study include its cross-sectional approach as it only analyzed one point in time and its small sample size, which lacked the ability for generalizability.

Kasparian et. al explored psychological responses and coping strategies among patients with malignant melanoma [2]. Malignant melanoma is a serious form of skin cancer with a significant psychological impact on patients. This review examines how individuals diagnosed with melanoma respond psychologically and the coping strategies they use to manage the associated distress. A diagnosis of melanoma often triggers a range of psychological responses, including anxiety, depression, fear of cancer recurrence, and concerns about body image. Patients may experience heightened levels of stress and emotional distress, particularly around the time of diagnosis and during treatment. Various coping strategies were identified, including problem-focused coping (e.g., seeking information and planning), emotion-focused coping (e.g., venting emotions and seeking social support), and avoidance coping (e.g., distraction or denial).

Effective coping strategies generally involved seeking social support and engaging in problem-solving, while avoidance coping was associated with poorer psychological outcomes. Psychological responses and coping strategies were influenced by factors such as age, gender, and disease stage. Younger patients tended to experience higher levels of distress compared to older individuals. Social support was highlighted as a critical factor in mitigating psychological distress. The review underscored the need for psychological interventions and support systems for melanoma patients, especially for those at higher risk of emotional distress [2].

Tailored interventions, including counseling and support groups, could help patients manage their anxiety and improve their overall quality of life. The review concluded that malignant melanoma has a profound psychological impact on patients, and coping strategies vary widely. Psychological interventions should be considered as part of comprehensive melanoma care to support patients' mental well-being. This article emphasized the importance of addressing the emotional and psychological aspects of melanoma alongside physical treatment [2].

Kovács et al. examined the role of social support in mitigating the depressogenic (depression-inducing) effects of low-dose interferon-alpha (IFN-α) treatment in melanoma patients. IFN-α is commonly used in the treatment of melanoma due to its immune-modulating effects. However, it has significant side effects, including inducing depression in patients. This study investigated how social support influences the development of depressive symptoms in melanoma patients undergoing low-dose IFN-α treatment. Low-dose IFN-α treatment was found to induce depressive symptoms in melanoma patients, consistent with previous findings that link IFN-α to mood disturbances. Depression was a notable side effect, although not all patients were equally affected. The study revealed that social support played a protective role against the development of depression during IFN-α treatment. Patients with strong social support networks from family, friends, or caregivers have significantly lower levels of depressive symptoms compared to those with weaker or limited social support. Patients were assessed using standardized depression rating scales before and during IFN-α treatment. Those with higher social support scores experienced less severe depressive symptoms, highlighting the buffering effect of social relationships on mental health during treatment [6]. As evidenced by the Beck Depression Inventory, high-supported patients expressed fewer depressive symptoms than those with low support (mean=5.71, SD=4.765; mean=5.65, SD=5.125, respectively; t=-0.072, p=0.943). The exact mechanism through which social support mitigates depression was not fully elucidated, but the authors suggest that emotional support, practical help, and a sense of belonging may reduce stress and enhance coping during the difficult treatment process. The findings underline the importance of screening for depression and enhancing social support networks for melanoma patients undergoing IFN-α therapy. Incorporating psychosocial interventions that strengthen social ties could be a valuable strategy for reducing depression risk during treatment. The study concluded that social support can significantly reduce the risk of depression in melanoma patients receiving low-dose IFN-α treatment. Given the mood-altering effects of IFN-α, integrating psychosocial support into patient care could improve mental health outcomes and overall quality of life for these patients. This research highlighted the critical role of social support in cancer care, particularly when patients are receiving treatments that may induce depression [6].

Dunn et. al detailed that patients employ various coping mechanisms, including seeking social support and engaging in problem-solving and distraction techniques. However, effectiveness varied, and not all patients have access to or use supportive resources [14]. Access to psychosocial support services, such as counseling or support groups, can help mitigate some of the psychological burdens. Supportive care interventions are essential for addressing mental health needs and improving quality of life. The review underscored the importance of integrating psychosocial care into the overall treatment plan for patients with advanced melanoma. This included regular screening for mental health issues and providing appropriate psychological support. Personalized care plans that address individual psychological needs and preferences are recommended to enhance patient outcomes. The systematic review concluded that patients with advanced melanoma experience significant psychosocial challenges, including high levels of distress, depression, and anxiety, which negatively affect their quality of life. Effective psychosocial support and tailored interventions are crucial for improving mental health outcomes and overall well-being in this patient population. This review highlighted the need for comprehensive care that not only addresses the physical aspects of advanced melanoma but also provides robust psychological and emotional support [14].

Interventions

Lynch et al. described a feasibility study about the Fear-Less program with the aim of reducing the fear of cancer recurrence in melanoma patients. The Fear-Less program is a stepped-care approach involving 61 melanoma patients currently receiving immunotherapy or targeted therapy for melanoma [9]. Patients either receive step 1 care, which consists of low-intensity self-help materials for self-management, or step 2 care, which consists of high-intensity in-person therapist cognitive behavioral therapy (CBT) based on early screening. The screening utilized both the Fear of Cancer Recurrence Inventory Short Form (FCRI-SF) and the Fear of Progression Questionnaire Short Form (FoP-Q-SF). The mean age of patients was 61.4 years old (SD=11.6), and the participants consisted of 41 (67%) male and 20 (33%) female individuals. Of the 27 patients offered self-help management, 24 (89%) patients accepted this intervention. Of the patients who accepted the self-management intervention, 21 patients completed the intervention. Thirteen (62%) patients out of the 21 patients who completed the intervention reported that they would recommend this style of self-management to others. This was evidenced by a reduction in FCRI-SF post-intervention (mean=16.90, SD=7.69) compared to pre-intervention (mean=17.67, SD=6.03) for patients who had undergone the self-management intervention. Twelve patients were referred for in-person therapy, which entailed step 2 care. Of these 12 patients, 11 (92%) patients accepted the referral to CBT. Following the referral, six (100%) patients completed the full five therapy sessions and recommended this style of in-person management to others. This was evidenced by the reduction in FCRI-SF and FoP-Q-SF post-intervention (mean=20.57, SD=6.35; mean=33.71, SD=7.87, respectively) compared to pre-intervention (mean=24.29, SD=4.19; mean=37.29, SD=8.56, respectively). Due to the positive feedback from patients, the authors suggested screening melanoma patients undergoing treatment for fear of cancer progression and offered early intervention options [9].

Fawzy et al. assessed the long-term effects of a brief, structured psychiatric intervention on survival and recurrence rates in 68 patients with malignant melanoma over 10 years [10]. The 68 patients were divided into two groups: control and experimental. The control group consisted of 17 male patients and 17 female patients and had a mean age of 39.3 years. The experimental group consisted of 16 male patients and 18 female patients and had a mean age of 45.7 years. The groups did not significantly differ in sex (p=0.81), Breslow depth (p=0.98), or tumor site (p=0.35); however, they did significantly differ in age (p=0.02) [10]. All groups had a diagnosis and surgical treatment of stage 1 melanoma. The control group did not have educational or psychological interventions, while the experimental group attended weekly meetings for psychological intervention. These meetings were 1.5 hours for six weeks and discussed topics such as health education, stress management, coping skills, and psychological support. After assessing the surviving patients at the 10-year follow-up, the experimental group's participation in the intervention had no effect on recurrence, although it was significant for survival (p=0.05) [10].

Discussion

The studies included in this narrative review have recognized that melanoma patients faced similar fears and changes in psychological well-being that were highly influenced by other factors such as age, gender, social support, status of baseline psychiatric conditions, and stage of diagnosis [2,6,8,9,11,13,14]. Patients who were diagnosed at a younger age had increased depression and anxiety. While female gender was associated with increased severity of depression and anxiety [10], male gender was associated with recurrence and poorer prognosis and outcomes [11]. Patients with pre-existing psychiatric conditions at baseline tended to have more struggles with treatment adherence leading to worse prognosis [15]. Studies suggested that this may be due to a lack of social support, decreased cognitive functioning, or feelings of hopelessness [2,6,8,13,15]. In terms of the stage of diagnosis, high levels of anxiety and depression were expressed by patients with early-stage melanoma and patients with advanced stages of melanoma (stage 3 and stage 4 melanoma) [12]. Patients with early-stage melanoma experienced a more rapid resolution of anxiety and depression post-excision [11] with improved mental health and quality of life [9] as survival extended for those patients who participated in psychological well-being interventions [10]. This contrasted with persistent depression and anxiety among patients with unresectable stage 3 and stage 4 melanoma during follow-up visits [12].

Practical Implications and Recommendations for the Psychological Well-Being of Melanoma Patients

As evidenced by these studies, there is a need for sustainable psychological intervention tailored specifically to the necessities and wishes of patients diagnosed with melanoma [2,9,13,14]. According to the studies, tailored interventions were found to be particularly crucial for patients diagnosed with advanced melanoma (stage 3 and stage 4 melanoma) [8,9,12,14]. Patients have shared high satisfaction with both self-management via psycho-educational resources and in-person psychotherapy sessions, as well as decreased fear of recurrence with both forms of intervention [9,16]. Overall, three aspects of psychological experiences helped improve and maintain the well-being of melanoma patients: (a) social support, (b) self-management, and (c) in-person therapy. It was evidenced that simultaneous implementation of these three aspects yielded the most perceived benefits for melanoma patients [16].

Social support: The most studied factor that sustained psychological well-being in melanoma patients was social support. Establishing social support early in the disease progress has yielded a decrease in anxiety and depression among melanoma patients [2,6,13]. Social support served as an emotional coping strategy that mitigated psychological distress for most patients with melanoma [2].

Self-management: A thorough read of psycho-educational resources such as booklets [13] served as the primary avenue for self-management in melanoma patients [6]. Resources on melanoma health education, coping skills, and stress management have served a beneficial role for patients with melanoma [1,7,6,13].

In-person therapy: In-person sessions in the form of CBT [9] and psychotherapy [16] have been shown to decrease the fear of cancer recurrence at follow-up visits, compared to the fear of cancer recurrence severity at baseline before psychological interventions. Tailored in-person therapy sessions that aligned with patients' goals allowed for discussions on skin self-examination, anticipation of medical appointments, and personal reflections on the quality of life with melanoma, which helped decrease the fear of cancer recurrence among patients [9,16].

Although these studies offered psychological well-being recommendations for melanoma patients, they largely overlooked the late-stage population who are not candidates for cancer excision. Given the significant psychological challenges faced by these patients, it is crucial to expand the literature focusing on this group. While the need for appropriate assessment tools for the psychological needs of melanoma patients has been highlighted [2], such a tool has yet to be developed. Additionally, there is a lack of focused quality-of-life measures, such as fatigue, cognition, and sleep [3]. Addressing these gaps in the literature could pave the way for more comprehensive care.

Limitations

This narrative review presents several limitations that should be acknowledged. First, the scope of the review was limited to studies published within the last 25 years, which may have excluded relevant older literature that could provide additional context or historical perspectives on the psychological experiences of melanoma patients. Second, the search was restricted to studies published in English, potentially omitting important findings from non-English publications. This language bias may have limited the comprehensiveness of the review, especially considering that melanoma is a global health issue. Third, the reliance on electronic databases such as Google Scholar, PubMed, and MEDLINE, while extensive, may have missed relevant studies not indexed in these databases or those published in less prominent journals. The exclusion of case reports and non-peer-reviewed studies, although aimed at ensuring the quality of the included studies, might have overlooked unique insights from less conventional research formats. In addition, the two-week window of database search time places potential limitations on the breadth of literature encountered.

Another limitation is the heterogeneity of the studies included. The variability in study designs, sample sizes, and assessment tools across the included studies complicates the direct comparison and synthesis of findings. This heterogeneity could contribute to an under- or overestimation of the psychological impact on melanoma patients. The review also largely focuses on anxiety, depression, and quality of life, with less emphasis on other psychological outcomes such as cognitive function, sleep disturbances, and fatigue. The limited number of studies addressing these broader psychological aspects indicates a gap in the literature and suggests the need for future research to explore these areas more comprehensively. Furthermore, the use of cross-sectional designs in many included studies poses a limitation as they capture data at a single point in time and do not provide insights into the longitudinal psychological trajectory of melanoma patients. Thus, it is unable to be determined in these studies if patients had a previous psychiatric history. Longitudinal studies are needed to better understand the evolution of psychological symptoms and the effectiveness of various coping strategies and interventions over time.

Lastly, the review is constrained by the inclusion of studies conducted in various countries with differing healthcare systems and cultural contexts, which may affect the generalizability of the findings. Cultural differences in the perception of illness and psychological well-being could influence the reported outcomes, thus limiting the applicability of the review's conclusions to broader populations.

Future Research

The current literature available on anxiety and depression experienced among melanoma patients offers insight into the psychological experiences of patients from the time of diagnosis to treatment and post-treatment follow-ups. Depression is well-documented in melanoma patients, although more research is needed to determine effective pharmacological treatments for this population. There also remains a pressing need for further research to explore other facets of the psychological experience of melanoma patients. Cognitive function, self-esteem, sleep disturbances, and social well-being are aspects that are often overlooked but are vital to understanding how advanced melanoma affects individuals in a more holistic manner. These areas are essential for providing a complete picture of a patient's experience, as such factors not only influence their mental health but also their ability to engage with treatment, quality of life, and overall outlook on life. To elucidate the unique struggles of melanoma patients, it may be helpful to compare a psychological aspect of diagnosis, such as depression, to other patient populations such as those with multiple sclerosis. Addressing underexplored dimensions in future research could lead to the development of more effective psychological support interventions, tailored to the unique needs of this population.

## Conclusions

As medical care becomes more specialized, being mindful of the importance of holistic, encompassing care is vital to treating all aspects of a patient. In those diagnosed with advanced, unresectable melanoma, anxiety and depression are common comorbidities that can be proactively accounted for in future treatment planning. The importance of future research to understand other aspects of the psychological experience of those diagnosed with stage 3 or stage 4 unresectable melanoma such as cognition, self-esteem, sleep, and many other factors cannot be understated. With a better psychological understanding of melanoma patients, healthcare providers will be able to better support their patients physically, mentally, and emotionally.
